# The prevalence, characteristics, and factors associated with purchasing Chinese herbal medicine among adults in Taiwan

**DOI:** 10.1186/s12906-017-1679-2

**Published:** 2017-03-27

**Authors:** Chun -Chuan Shih, Lu-Hsiang Huang, Chun-Chieh Yeh, Hsin-Long Lane, Chang-Ju Hsieh, Chin-Chuan Tsai, Li-Wei Lin, Ta-Liang Chen, Chien-Chang Liao

**Affiliations:** 1 The School of Chinese Medicine for Post-Baccalaureate, I-Shou University, Kaohsiung, Taiwan; 2 Program for the Clinical Drug Discovery from Botanical Herbs, Taipei Medical University, Taipei, Taiwan; 3 Taipei Chinese Medical Association, Taipei, Taiwan; 4 Quality of Traditional Chinese Medicine and Pharmacy Medical Association, Taipei, Taiwan; 5 Clinical Informatics and Medical Statistics Research Center, Chang Gung University, Taoyuan, Taiwan; 6 Department of Surgery, China Medical University Hospital, Taichung, Taiwan; 7 Department of Surgery, University of Illinois, Chicago, IL USA; 8 Department of Chinese Medicine, Changhua Christian Hospital, Changhua, Taiwan; 9 Department of Anesthesiology, Taipei Medical University Hospital, 252 Wu-Xing Street, Taipei, 110 Taiwan; 10 Department of Anesthesiology, School of Medicine, College of Medicine, Taipei Medical University, Taipei, Taiwan; 11 Anesthesiology and Health Policy Research Center, Taipei Medical University Hospital, Taipei, Taiwan; 12 School of Chinese Medicine, College of Chinese Medicine, China Medical University, Taichung, Taiwan; 13 Department of Anesthesiology, Shuan Ho Hospital, Taipei Medical University, New Taipei City, Taiwan

## Abstract

**Background:**

The objective of this study was to investigate the prevalence and factors associated with purchasing Chinese herbal medicine (CHM) without a physician’s prescription among adults.

**Methods:**

Using data from the 2005 National Health Interview Survey and National Health Insurance, we identified 16,756 individuals aged 20 years and older. Socio-demographic factors, lifestyle, medical services utilization and health behaviors were compared between people with and without a history of purchasing CHM by calculating adjusted odds ratios (ORs) and 95% confidence intervals (CIs) in a multiple logistic regression analysis.

**Results:**

The one-month prevalence of purchasing CHM without a physician’ prescription was 5.2% in Taiwan. People more likely to purchase CHM included people aged ≥70 years (OR 2.84, 95% CI 2.03-3.99), women (OR 1.28, 95% CI 1.11-1.48), non-indigenous people (OR 2.61, 95% CI 1.29–5.30), and people with an illness not receiving medical care (OR 2.69, 95% CI 2.19-3.31).

**Conclusion:**

The prevalence of purchasing CHM without a physician’s prescription is high in Taiwan and is correlated with factors such as socio-demographics, disease history, and behaviors surrounding the utilization of medical care.

## Background

In the past ten years, the use of complementary alternative medicine (CAM) among children and adults in Asian and Western countries has been rising [1–9]. Traditional Chinese medicine (TCM) is one of the most popular forms of CAM and is commonly used by people in Taiwan [4, 5]. The one-year prevalence of TCM use was as high as 28.4% in 2001 [5, 10]. Increasing use of TCM has also been reported in Western countries [11–13]. The use of and expenditures associated with CAM among adults in the United States increased substantially between 1990 and 1997 [2].

Although the outpatient payments for both TCM and Western medicine have been covered by Taiwan’s National Health Insurance since 1995, the purchase of non-prescription medicine in pharmacies is still a common occurrence [12]. With the advent of several reports regarding the nephrotoxic and hepatotoxic effects associated with CHM use [14–16], the purchase of CHM without a physician’s prescription has become an important health issue [17]. Purchasing CHM without a physician’s prescription may lead to adverse effects from either the CHM itself or because of interactions between the CHM and biomedical medicines [18–22]. A previous study reported that patients with multiple chronic diseases are the major purchasers of TCM products not covered by Taiwan’s National Health Insurance (NHI) program [23]. However, some previous studies provide limited information and are plagued by limitations such as small sample size and selection bias [4, 11–13].

Furthermore, there is a paucity of information describing the characteristics of people who purchase CHM without a physician’s prescription in Taiwan. Using data from the 2005 National Health Interview Survey (NHIS) and NHI, we conducted a cross-sectional study to investigate the prevalence and factors associated with the purchase of CHM without a physician’s prescription among adults in Taiwan.

## Methods

### Study design

In 2005, the National Health Research Institute and the Bureau of Health Promotion of Taiwan conducted the National Health Interview Survey (NHIS) using face-to-face questionnaire interviews. The population of Taiwan is approximately 23 million and is distributed throughout 7 cities and 18 counties. The 2005 NHIS included a representative sample of 24,726 interviews from the non-institutional population. The interviewees were all residents, and each interview was performed in the subject’s home. All subjects interviewed were selected from the household census. With the standardized face-to-face questionnaire interview, the NHIS used a multistage, stratified systematic sampling scheme to interview a nationally representative sample of the population of Taiwan. The response rate was 80.6% for individual subjects. At the end of the NHIS, the participants were asked for permission to access their NHI records for research purposes. All study participants signed the informed consent to link their information with the NHI claims data to retrieve information on medical service use in 2005. This study analyzed 16,756 study participants aged 20 years and older. Our study was evaluated and approved by the Joint Institutional Review Board of E-DA Hospital (EDA-JIRB-2017002).

### Data collection

Both NHIS data and NHI claims data from 2005 were used in this study. With the written informed consent of eligible NHIS participants that allowed the linking of their NHI data, the 2005 NHIS data were linked to the 2005 NHI claims data. Information about prevalence and frequency of TCM and Western medicine use were drawn from the 2005 NHI data. The core question in this study was “Have you ever purchased Chinese herbal medicine for yourself or your family members without a physician’s prescription or advice in the past month?” Socio-demographic data (such as age, gender, education, occupation, family income, ethnicity, religion and marital status) and data on folk therapy, regular health checkups and unhealthy lifestyle (including smoking, alcohol use, and betel nut chewing) were derived from the NHIS data.

### Definitions and measures

In Taiwan, TCM includes Chinese herbal medicine, acupuncture, moxibustion, bone reduction (setting a broken bone without surgery), traditional trauma treatment, traditional dislocation treatment, traditional fracture treatment, Tuina (massage and kneading), Baguan (cupping or vacuum bottle therapy), and other therapies. TCM practitioners are registered TCM physicians and practice in a hospitals or clinics. TCM in Taiwan is legal, and TCM physicians can advertise the medical benefits of TCM according to medical law in Taiwan [5]. Chinese herbal medicine is the theory of traditional Chinese herbal therapy, which was the majority of treatments in TCM. Acupuncture is one of forms of TCM in which very thin needles are inserted into some specific points of body. Moxibustion is also one of forms of traditional Chinese medicine therapy which burning dried mugwort on some specific points of the body.

Folk therapy use is defined as the utilization of folk therapy within the past month. The types of folk therapy studied include Gua Sha (skin scraping), Tuina (massage and kneading), Baguan (vacuum bottle therapy), bone setting, spine alignment, Qigong, divination, written charms, shaman consultation, talismans, incense ash, and other related therapies. The difference between TCM and folk therapy lies in their legality. Legislation from the Department of Health in Taiwan has declared that folk therapy practitioners cannot claim any medical benefits from folk therapy. Folk therapy practitioners do not have certifications or legal licenses, and they do not work in clinical settings in Taiwan. For the Western countries, TCM and folk therapy were included in the CAM. However, TCM and folk therapy were different in Taiwan because their legality.

We calculated the density of TCM physicians (TCM physicians/10,000 persons) using the number of TCM physicians per 10,000 persons for each of the administrative units. The first, second, and third tertiles were considered areas of low, moderate, and high TCM physician density, respectively [5, 24]. A high prevalence of cigarette smoking, alcohol drinking, and betel quid chewing has been found in Taiwan [25]. People with a habit of smoking tobacco (included some periods of smoking in a lifetime, or less than 5 packs in a lifetime, or more than 5 packs in a lifetime), drinking alcohol (current drinkers and any type of alcohol drinks) and/or chewing areca were considered to be engaging in unhealthy lifestyles that are associated with cancer and other diseases [4, 5].

### Statistical analysis

In the study, chi-square tests were used to compare the difference in socio-demographic factors, lifestyles, and medical care behaviors between people with and without CHM use. Fisher’s exact test was used when the sample size was small. We used multivariate logistic regression analysis to analyze the factors of TCM and Western medicine use associated with CHM and estimated the odds ratio (OR) and corresponding 95% confidence interval (CI). These covariates included age, gender, family income, ethnicity, marital status, unhealthy lifestyle, folk therapy and density of TCM physicians. For each covariate, we assigned a predicted score as a risk index according to the significant adjusted OR, and the predicted score is proportional to the OR. Purchasing predictive score was defined as follows: when 1.0 ≤ OR <1.5, the purchasing predicted score was 1, when 1.5 ≤ OR <2.0, the purchasing predicted score was 2, when 2.0 ≤ OR <2.5, the purchasing predicted score was 3, and when 2.5 ≤ OR <3.0, the purchasing predicted score was 4. All analyses were performed using Statistical Analysis Software (SAS), version 9.2 (SAS Institute Inc., Cary, North Carolina, U.S.A.). A two-sided *p*-value less than 0.05 was considered significant.

## Results

Among the 16,756 participants aged 20 years and older (Table 1), the one-month prevalence of purchasing CHM was 5.2%. A higher percentage of older people (≥70 years) purchased CHM than other age groups, and more females than males purchased CHM. A higher percentage of participants who were non-indigenous and who were ill with or without medical care in the past six months compared with those without illness purchased CHM. The two groups were comparable in terms of marital status, employment status, family income and educational level. In the multivariate logistic regression analysis, the following individuals were more likely to purchase CHM: females, people aged 40-49, 50-59 or 60-69 years old, non-indigenous people, people who had one or two or more unhealthy lifestyles, people who lived in areas where the density of practitioners was high and people who were ill with or without medical care in the past six months.

**Table 1:** Characteristics of people with and without purchasing CHM

	Purchase of CHM							
	No (*N* = 15,889)		Yes (*N* = 867)			Multivariate adjusted		
	n	(%)	n	(%)	*p*-value	OR	(95% CI)^c^	Score^d^
Age, years								
20–29	3561	(97.8)	81	(2.2)	<0.0001	1.00	(reference)	0
30–39	3331	(95.8)	145	(4.2)		1.87	(1.42–2.48)	2
40–49	3469	(93.8)	230	(6.2)		2.71	(2.06–3.55)	4
50–59	2394	(93.0)	179	(7.0)		2.82	(2.10–3.77)	4
60–69	1566	(93.0)	118	(7.0)		2.80	(2.02–3.88)	4
≥ 70	1568	(93.2)	114	(6.8)		2.84	(2.03–3.99)	4
Gender								
Male	8166	(95.5)	382	(4.5)	<0.0001	1.00	(reference)	0
Female	7723	(94.1)	485	(5.9)		1.28	(1.11–1.48)	1
Occupation								
White collar	5821	(92.5)	276	(7.5)	0.02	-	-	-
Blue collar	5828	(93.9)	336	(6.1)		-	-	-
Others	4284	(93.4)	255	(6.6)		-	-	-
Education, years								
0	1158	(92.8)	90	(7.2)	<0.0001	1.25	(0.90–1.73)	1
1–9	5350	(93.0)	404	(7.0)		1.46	(1.18–1.80)	1
10–12	4555	(95.9)	194	(4.1)		1.03	(0.83–1.27)	1
≥ 13	4826	(96.4)	179	(3.6)		1.00	(reference)	0
Family income, NTDs								
< 30,000	4106	(93.5)	287	(6.5)	<0.0001	-	-	-
30,000–200,000	11,360	(95.3)	556	(4.7)		-	-	-
> 200,000	423	(94.6)	24	(5.4)		-	-	-
Marital status								
Married	10,115	(94.2)	626	(5.8)	<0.0001	-	-	-
Unmarried	3965	(97.5)	101	(2.5)		-	-	-
Others	1809	(92.8)	140	(7.2)		-	-	-
Ethnicity								
Non-indigenous	15,532	(93.2)	857	(6.8)	0.012	2.61	(1.29–5.30)	4
Indigenous	401	(96.1)	10	(3.9)		1.00	(reference)	0
Density of physicians^a^								
Low	5657	(94.3)	342	(5.7)	0.0002	1.16	(0.97–1.37)	1
Moderate	5351	(95.3)	365	(4.7)		1.38	(1.16–1.65)	1
High	4881	(94.9)	260	(5.1)		1.00	(reference)	0
Unhealthy lifestyles^b^								
None	7824	(93.5)	442	(5.4)	0.4785	-	-	-
One	3575	(93.0)	181	(4.8)		-	-	-
Two or three	4490	(93.0)	244	(5.2)		-	-	-
Medical care in past 6 months								
Without illness	4974	(97.0)	156	(3.0)	<0.0001	1.00	(reference)	0
Illness with medical care	7409	(94.2)	453	(5.8)		1.83	(1.52–2.21)	2
Illness without medical care	3506	(93.2)	258	(6.8)		2.69	(2.19–3.31)	4

*CHM* Chinese herbal medicine, *TCM* traditional Chinese medicine

^a^Physicians/km^2^. ^b^Including alcohol drinking, smoking and betel nut chewing. ^c^Adjusted for all covariates in this table. ^d^Predicted scores: 1.0 ≤ OR <1.5 predicted score = 1, 1.5 ≤ OR <2.0 predicted score = 2, 2.0 ≤ OR <2.5 predicted score = 3, 2.5 ≤ OR <3.0 predicted score = 4, 3.0 ≤ OR <3.5 predicted score = 5, 3.5 ≤ OR < 4.0 predicted score = 6

After adjustment, those more likely to purchase CHM included individuals who purchased biomedical medicine without a prescription and those who had received emergency care, hospital care or outpatient care in the past year (Table 2).

**Table 2:** Medical care and western medicine use for people with and without purchasing CHM

	Purchase of CHM						
	No (*N* = 15,889)		Yes (*N* = 867)			Multivariate	
	n	(%)	n	(%)	*p*-value	OR	(95% CI)^b^
Purchase of biomedical medicine^a^							
No	13,172	(95.4)	632	(4.6)	<0.0001	1.00	(reference)
Yes	2717	(92.0)	235	(8.0)		1.60	(1.36–1.88)
Emergency care in past year							
No	13,721	(95.0)	723	(5.0)	0.0137	1.00	(reference)
Yes	2168	(93.8)	144	(6.2)		1.17	(0.97–1.42)
Hospitalized care in past year							
No	14,265	(95.0)	759	(5.0)	0.0352	1.00	(reference)
Yes	1624	(93.8)	108	(6.2)		1.09	(0.87–1.35)
Outpatient care by WM in past year^a^							
No	4893	(95.1)	255	(4.9)	0.3900	1.00	(reference)
Yes	10,996	(94.7)	612	(5.3)		1.09	(0.93–1.27)
Frequency of outpatient care by WM in past year							
None	4899	(95.1)	255	(4.9)	<0.0001	1.00	(reference)
Low	3739	(96.2)	149	(3.8)		0.92	(0.74–1.14)
Moderate	3645	(94.7)	203	(5.3)		1.14	(0.94–1.38)
High	3606	(93.3)	260	(6.7)		1.18	(0.98–1.41)
Expenditure of WM in past year							
None	4899	(95.1)	255	(4.9)	<0.0001	1.00	(reference)
Low	3681	(96.2)	147	(3.8)		0.92	(0.74–1.14)
Moderate	3619	(94.5)	210	(5.5)		1.18	(0.97–1.43)
High	3690	(93.5)	255	(6.5)		1.14	(0.95–1.38)

*CHM* Chinese herbal medicine, *WM* Western medicine

^a^Purchase biomedical medicine without a physician’s prescription. ^b^Adjusted for age, gender, education, ethnicity, density of TCM physicians, and medical care in the past 6 months in multiple logistic regression

The use of folk therapy and TCM use in the past year were associated with purchasing CHM (Table 3). In addition, the frequency and expenditure of TCM use were also factors associated with the purchase of CHM. In Table 4, we divided the predictive scores into six groups (0-4, 5, 6, 7, 8, and 9), and the 0-4 group was considered as the comparison group in the analysis. The predictive scores 5, 6, 7, 8, 9 were highly associated with purchasing CHM.

**Table 3:** Medical care visit of TCM for people with and without purchasing CHM

	Purchase of CHM						
	No (*N* = 15,889)		Yes (*N* = 867)			Multivariate	
	n	(%)	n	(%)	*p*-value	OR	(95%CI)^a^
Use of TCM in past year							
No	11,952	(95.1)	610	(4.9)	0.0070	1.00	(reference)
Yes	3937	(93.9)	257	(6.1)		1.26	(1.08–1.47)
Use of TCM in past year							
No	11,952	(95.1)	610	(4.9)	0.0071	1.00	(reference)
1 times	1192	(94.2)	73	(5.8)		1.26	(0.98–1.63)
2 times	673	(94.5)	39	(5.5)		1.13	(0.81–1.59)
≥ 3 times	2072	(93.5)	145	(6.5)		1.29	(1.07–1.56)
Use of folk therapy							
No	14,872	(95.2)	743	(4.8)	<0.0001	1.00	(reference)
Yes	1012	(89.2)	123	(10.8)		2.23	(1.82–2.74)
Visits of TCM in past year							
No	11,952	(95.1)	610	(4.9)	0.0073	1.00	(reference)
Low	1865	(94.3)	112	(5.7)		1.22	(0.98–1.50)
Moderate	1119	(93.4)	79	(6.6)		1.35	(1.06–1.73)
High	953	(93.5)	66	(6.5)		1.23	(0.94–1.60)
Expenditure of TCM in past year							
No	11,952	(95.1)	610	(4.9)	0.0152	1.00	(reference)
Low	1276	(94.2)	78	(5.8)		1.25	(0.97–1.59)
Moderate	1320	(93.7)	89	(6.3)		1.31	(1.04–1.66)
High	1339	(93.8)	89	(6.2)		1.20	(0.95–1.52)

*CHM* Chinese herbal medicine, *TCM* traditional Chinese medicine

^a^Adjusted for age, gender, education, ethnicity, density of TCM physicians, and medical care in the past 6 months in the multivariate logistic regression

**Table 4:** Predictive scores of purchasing CHM without physician’s prescription

		Purchasing CHM			
	n	Events	Prevalence	OR	(95% CI)
Purchasing predictive scores					
0–4	8437	255	3.00%	1.00	(reference)
5	1683	98	5.82%	1.99	(1.57–2.53)
6	1751	115	6.56%	2.27	(1.80–2.86)
7	1671	110	6.58%	2.27	(1.80–2.86)
8	1685	156	9.26%	3.29	(2.67–4.40)
9	1493	133	8.91%	2.87	(2.31–3.57)

*CHM* Chinese herbal medicine, *CI* confidence interval, OR, odds ratio

## Discussion

Using the data from the NHIS and NHI, we found that the one-month prevalence of purchasing CHM without a physician’s prescription in adults was 5.2%. Furthermore, the likelihood of purchasing CHM was associated with socio-demographic factors, disease history, and utilization of medical care.

The results of this study showed that women and middle-aged individuals (30-59 years old) have a higher prevalence of purchasing CHM. These findings are similar to results from previous investigations that reported a correlation between age and gender and self-medication [26–28]. Compared with the elderly, younger people are more knowledgeable about medical care and have a better attitude towards and practice of using medical services [29]. A previous study also suggested that young people are more likely to seek treatment to improve their well-being and disease-related symptoms than older people [30]. Our results suggest that because middle-aged people are generally the primary caregivers in a family, they may be more likely to purchase medicine for their children or parents. In this study, gender was also found to be an important factor associated with purchasing CHM. Women may be more willing to address their health problems by trying multiple therapies not covered by the insurance system. In addition, social networking makes it easier for women to access CHM than men [31].

In the present study, we found that participants who were non-indigenous and living in areas with a moderate density of physicians had a higher likelihood of purchasing CHM. In general, residents in urban areas have more access to medical services than rural residents because more physicians practice in cities than in rural areas [32]. Therefore, people living in areas with a high density of TCM physicians have more opportunities to access conventional and unconventional therapies [8]. Explanations of why ethnicity differences exist are complex. Ethnicity covers many factors such as culture, nationality and religion and is also closely associated with socioeconomic status, which affects the accessibility to care, health status, and other predictors of conventional healthcare use [27, 33]. Therefore, it is important to determine if ethnic differences in folk therapy use emerge or persist when several of these predictors are considered simultaneously. Participants with a greater number of unhealthy behaviors were also more likely to use folk therapy. Shih et al. [12] suggested that individuals who engage in more unhealthy behaviors may consider folk therapy as a form of preventive medicine that can protect their health.

Many people choose to use an alternative therapy based on a recommendation from someone who has used the therapy and has been satisfied with the results. These referrals come mainly from family members, friends, acquaintances, and co-workers [11]. In addition, when an individual has more resources such as money, time, convenience, and access to TCM, the individual’s behavioral intention will be stronger. Previous studies have shown that income level is an important factor influencing people to seek alternative care [12]. According to a British study, older people have fewer financial resources to pay for private CAM [14]. Similarly, CAM users tend to have moderate to high incomes [15]. Some studies have reported that most unconventional medicine users are older people with high incomes [16, 18, 23]. We also found that older people purchase TCM products more often than young people, although the influence of personal income on TCM purchasing behavior was not significant. One possible reason that older people’s income did not predict purchasing behavior is that the influence of usable resources on behavioral intention should take into consideration not only personal income but also the total family income and other various enabling components. For example, older people have more time and ability to make decoctions from TCM herbs. Several decades ago, TCM was the main therapy in Taiwan, so most senior people have experience making decoctions from TCM herbs. Older people also tend to have more free time. As a result of these factors, the TCM purchasing behavior of the elderly is higher than that of younger individuals.

This study has several limitations. First, recall bias may exist because our data are based on self-reported questionnaires. Second, due to the cross-sectional study design, we could not provide information about whether purchasing CHM without a physician’ prescription is increasing or decreasing over time. Additionally, the causal relationship between purchasing CHM and associated factors cannot be confirmed in this cross-sectional study. Third, because purchasing CHM is a dichotomous variable, information on the dosage, frequency and expenditure is not available.

## Conclusions

In conclusion, purchasing CHM without a physician’s prescription in adults is common in Taiwan and is associated with socio-demographics, disease history, and utilization of medical care. Importantly, this study may alert TCM physicians that purchasing CHM without a physician’s prescription is a serious problem in Taiwan and requires considerable attention.

## Abbreviations

CAM: complementary alternative medicine
CHM: Chinese herbal medicine
CI: confidence interval
NHI: National Health Insurance
NHIS: National Health Interview Survey
OR: odds ratio
TCM: traditional Chinese medicine
